# Chinese medicine as comprehensive therapy for psoriasis: A case report

**DOI:** 10.1097/MD.0000000000040747

**Published:** 2024-11-29

**Authors:** Lingjuan Liu, Miao Li, Minhua Hu, Zhilin Ge, Xiulan Dong

**Affiliations:** a The First Affiliated Hospital of Guangzhou University of Chinese Medicine, Guangzhou, China; b Guangzhou University of Chinese Medicine, Guangzhou, China.

**Keywords:** case report, psoriasis, traditional Chinese medicine

## Abstract

**Rationale:**

Psoriasis is an immune-mediated, chronic, relapsing, inflammatory disease induced by a combination of genetic and environmental factors. It can be comorbid with other systemic diseases and severely affects patient’s quality of life. This case report describes the therapeutic role of traditional Chinese medicine (TCM) in patient with psoriasis.

**Patient concerns:**

The 32-year-old male patient exhibited symptoms of increased dandruff and recurrent erythematous scales on various body regions for nearly a year. At the same time, he had multisystemic metabolic abnormalities and psychiatric disorders.

**Diagnosis:**

Severe plaque psoriasis.

**Interventions:**

The patient was treated with oral Chinese herbal medicine only.

**Outcomes:**

Following TCM treatment, the patient demonstrated significant improvement in his skin condition and various metabolic-related indexes.

**Lessons:**

TCM is involved in the harmonization of yin and yang, regulation of qi and blood, dispersion of pathogenic wind, cooling of blood, and alleviation of blood stasis. From a modern medical perspective, TCM prescriptions may address skin inflammation by restoring physiological functions and enhancing immune response. This case study illustrates the efficacy of TCM in treating skin disorders and enhancing the body’s internal environment.

## 1. Introduction

Psoriasis is recognized by WHO as a “chronic, noncommunicable, painful, disfiguring, and disabling disease for which there is no cure.” At present, the estimated global prevalence reaches 3%.^[1]^ The multifaceted etiology of psoriasis encompasses genetic predisposition, immune dysregulation, and environmental influences.^[2,3]^ Exposure to external triggers, such as trauma, infection, and psychological stress, in conjunction with genetic susceptibility, can provoke the release of pro-inflammatory mediators by immune cells, including interleukin-17 and interleukin-23, and so on. These inflammatory factors will lead to the formation of an inflammatory environment in the skin, resulting in excessive proliferation of keratin-forming cells, which ultimately leads to hyperkeratosis of the epidermal tissues.^[4]^ Its typical clinical manifestations are localized or widespread scaly erythema or plaques, which may be accompanied by snow-white scaly flakes. Furthermore, certain academics have proposed the notion that individuals with psoriasis exhibit a higher susceptibility to metabolic disorders compared to the general populace, including insulin resistance, atherosclerosis, dyslipidemia, arterial hypertension, and cardiovascular disease.^[5,6]^ These comorbidities significantly diminish the patient’s quality of life and impose a substantial psychosocial burden.^[7,8]^

In recent years, psoriasis treatment has advanced, with topical therapies for mild cases and systemic oral treatments for moderate to severe cases.^[9]^ Mild psoriasis is usually managed with topical corticosteroids or vitamin D derivatives to reduce inflammation and scaling.^[10]^ Topical treatments are often insufficient for moderate to severe psoriasis, requiring systemic therapies such as acitretin, methotrexate, or cyclosporine are required for treatment.^[11]^ Currently, biologics are widely used for moderate to severe plaque psoriasis, including tumor necrosis factor inhibitors and interleukin inhibitors. Some psoriasis patients develop conditions such as eczema, atopic dermatitis, or urticaria after receiving biologic treatment, which limits the use of biologics and hinders adequate treatment for some patients with moderate to severe psoriasis.^[12]^ Moreover, long-term use of these treatments often leads to adverse effects.^[13,14]^ Immunosuppressants can raise infection risks, harm liver and kidney function, and cause other systemic issues, requiring regular health monitoring. Beyond the associated health risks, the substantial cost of treatment imposes a significant economic burden on patients. Additionally, some patients may experience paradoxical eczema events and severe infections of varying levels and types while receiving different biologics.^[15–17]^ Despite advancements in treatment, a definitive cure for psoriasis has yet to be discovered.^[18]^

Traditional Chinese medicine (TCM) theory categorizes psoriasis into 5 types: blood heat, blood drying, blood stasis, damp heat, and pyretic toxin. The clinical application of TCM (the intervention used in this case report was Chinese herbal medicines.) in treating psoriasis has shown promising potential.^[19]^ Previous research has been mainly basic research. Experiments have shown that TCM prescription can effectively alleviate skin psoriasis-like changes in mice.^[20]^ Various Chinese herb extracts, such as oxymatrine, have demonstrated efficacy in alleviating epidermal cell proliferation and apoptosis in severe plaque psoriasis.^[21,22]^ In view of the chronic and recurring characteristics of psoriasis, which is induced by the combined effect of genetics and environment, animal experiments still have certain limitations, such as fewer environmental interference factors and a short research period, so the thoroughness of treatment cannot be reflected. Long-term clinical follow-up is an important part of assessing the efficacy of drugs. By integrating knowledge of psoriasis in TCM with the etiology, pathogenesis, and pathological basis of Western medicine, we have developed a TCM treatment protocol that has demonstrated positive therapeutic outcomes.

## 2. Case presentation

### 2.1. Basic information about the patient

The 32-year-old male patient has experienced an abnormal increase of dandruff since August 2022, with recurrent episodes of erythematous scales on the face, chest, back, abdomen, and limbs, which made him feel inferior and depressed. According to his typical clinical symptoms, he was diagnosed with psoriasis by the Institute of Dermatology in Guangzhou, China. The patient is a front-line worker at the hospital pharmacy window. Due to his poor skin condition, the patients suspected him of having a contagious disease and filed multiple complaints against him in the hospital. Therefore, the Superior leadership has had several conversations with him, which added to his frustration.

The patient tried several treatments within 10 months. When he felt itching, he externally applied Purple Dragon ointment (an ancient dermatological preparation) and corticosteroids. When the itching was controlled, he moisturized with Vaseline. However, no obvious improvement was observed in this way. After the above treatment, the patient’s rash not only did not decrease, but was widely distributed throughout most of the skin of the body. Given the severity of his symptoms, he was recommended to seek treatment with immunosuppressive agents and biological agents by the Guangzhou Dermatitis Preventive Institute at the beginning of May 2023. The patient fortuitously encountered my teacher, a firm believer in TCM, who emphatically recommends treating with TCM and not using immunosuppressants for the time being considering the drugs’ risks. The patient presented at the clinic with a willingness to give it a try. During the course of Chinese herbal treatment, the patient also sought consultation at the Guangzhou Dermatitis Preventive Institute. Following an assessment by a professional dermatologist, it was determined that the patient’s skin condition had improved. Consequently, the patient was advised to continue the treatment regimen exclusively with Chinese herbal medicine.

### 2.2. TCM treatment and improvement of skin condition

**Figure 1.**
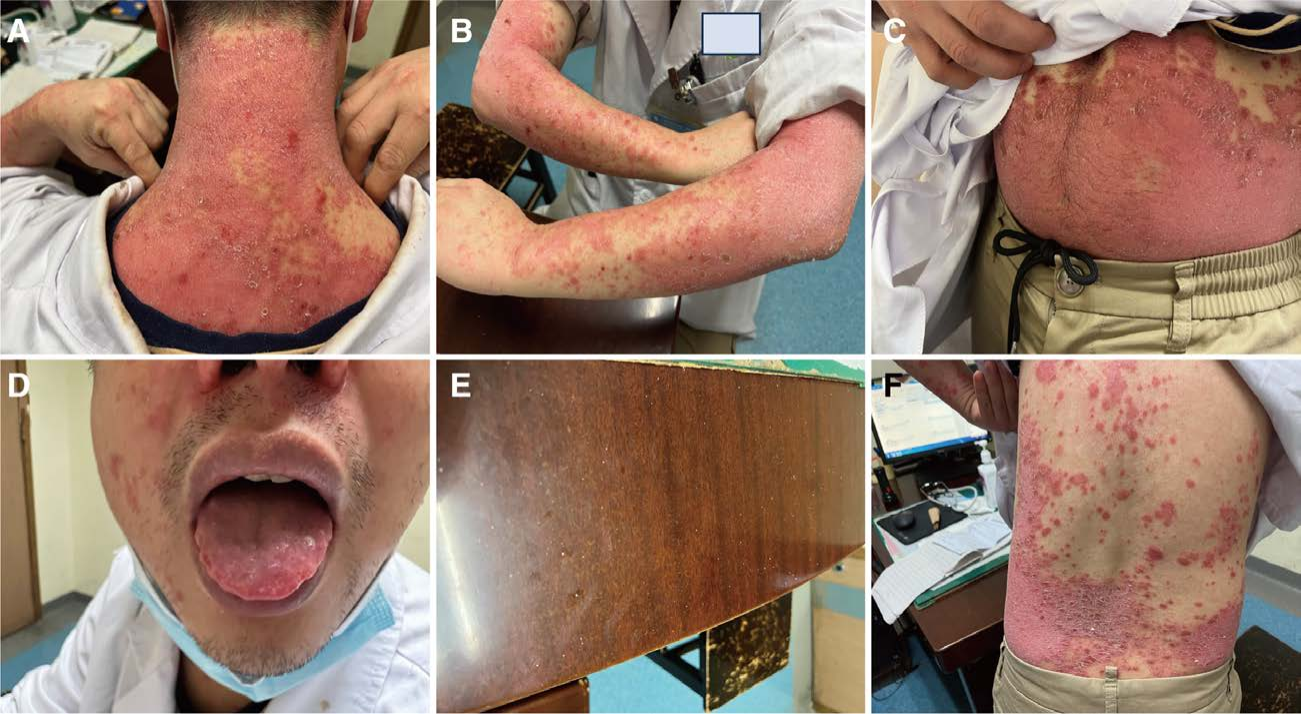
Patient’s skin condition at the first visit. (A–C and F) Skin condition. (D) Tongue image. (E) The desktop exhibited snow-white scales and peeling skin.

During the initial visit on May 24, 2023, it was observed that the majority of the patient’s skin exhibited extensive scarlet plaques, along with symptoms of dryness, itching, and desquamation. The desktop of the examination room where the patient once stayed exhibited a layer of snow-white scales and peeling skin (Fig. 1A–F). The patient exhibited a positive Auspitz sign. He was often under extreme stress, both physically and psychologically. He had a rather red tongue with teeth marks. According to the patient’s symptoms, tongue image, and pulse signs, we considered that TCM pathogenesis can be divided into 3 aspects, including the imbalance of Yin and Yang, excessive toxic heat, and pathogenic wind. In TCM, it is considered that the patient’s rash with a crimson hue and pruritus is interpreted as a manifestation of Yang Qi floating upward, making it difficult to descend and causing intense toxic heat in the upper burner. The variability in the location of the itching, affecting areas such as the head, limbs, chest, abdomen, and back, corresponds with the properties of wind pathogens, recognized for their propensity to move freely and change frequently. Consequently, the diagnosis is classified as “pathogenic wind.” However, the patient’s extremities and abdomen exhibit a lower temperature than normal, and the stool had a loose texture, suggesting the presence of cold-dampness in the lower burner according to TCM. This clinical presentation supports a diagnosis of imbalanced Yin and Yang, characterized by an excess of heat in the upper body and cold in the lower body, which is a significant pathological mechanism of the patient.

In this treatment, classical Chinese medicine prescriptions, specifically WumeiWan (WMW), were utilized to address the deficiency of Yang Qi, harmonize Yin and Yang at their origin, and invigorate the circulation of qi and blood (Table 1B). Additionally, skin diseases are always difficult to cure without endless toxins. In cases of severe blood-heat syndrome, high doses of Simiao Yongan Decoction were administered to cool the blood and remove toxins, exhibiting properties akin to anti-inflammatory medications (Table 1A). Lonicerae Japonicae Flos and Figwort Root are monarch herbs in Simiao Yongan Decoction, which play the main therapeutic role in the main disease or main syndrome. The manifestation of common skin symptoms, such as pruritus and erythema, can be attributed to the characteristic of wind, which tends to move and change. So, Chinese herbs were utilized to dispel pathogenic wind and relieve itching (Table 1C). After a soaking process lasting approximately 30 minutes, Chinese herbs are decocted in water for 30 to 60 minutes. Patient is instructed to consume 1 dose of herbal medicine per day, divided into 2 decoctions. Each decoction was boiled to 100 to 150 mL.

**Table 1:** Main prescription of traditional Chinese medicine treatment.

A	Lonicerae Japonicae Flos 50 g, Figwort Root 50 g, Angelicae Sinensis Radix 30 g, Licorice 10 g.
B	Mume Fructus 30 g, Phellodendri Amurensis Cortex 15 g, Zanthoxyli Pericarpium 5 g, Cinnanmomi Cortex 3 g, Aconiti Lateralis Radix Praeparata 10 g, Zingiberis Rhizoma 10 g.
C	Saposhnikoviae Radix 10 g, Schizonepetae Spica 15 g, Radix Rehmanniae Recen 30 g, Amomum Aurantiacum 10 g.
D	Hedysarum Multijugum Maxim 30 g, Radix Bupleuri 10 g, Schisandrae Sphenantherae Fructus l5 g.

**Figure 2.**
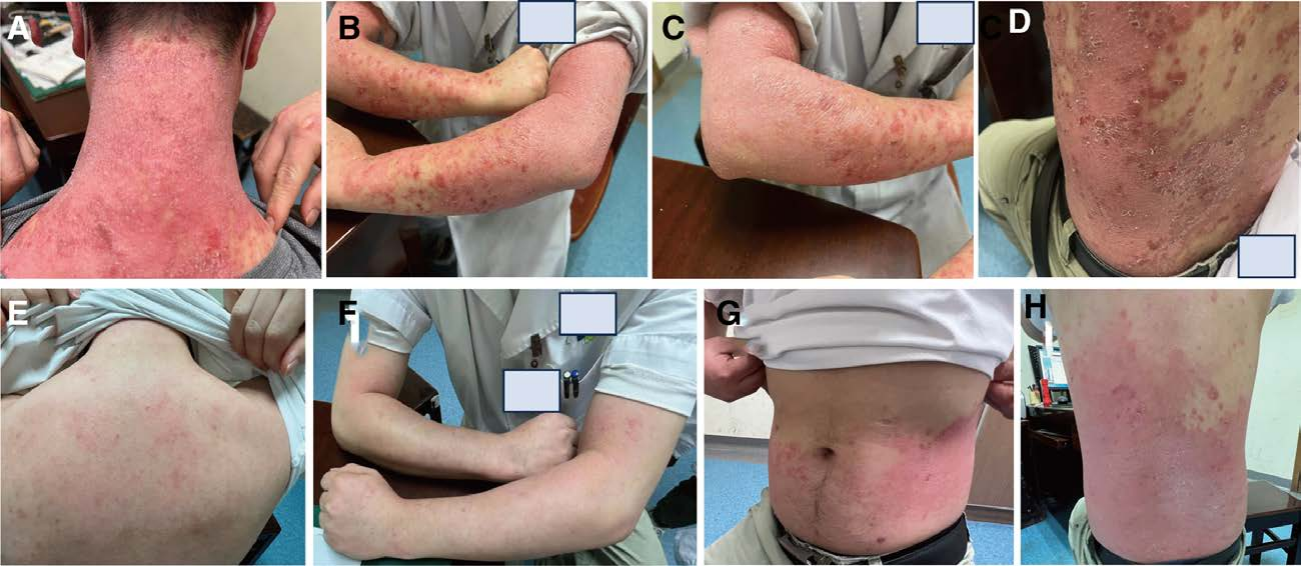
Patient’s skin condition at the second (A–D) and third visits (E–H).

During the second visit on May 31, 2023, the patient experienced alleviation of his pruritic skin symptoms, with a reduction in the color of his skin lesions and the emergence of normal skin in between (Fig. 2A–D). There is an old saying in TCM theory that the prescription should not be altered when effective. So, we continued with the last prescription and made minor adjustments based on this situation. The patient exhibited wiry pulses on the left hand and weak pulses on the right hand. They were the manifestations of liver qi stagnation and deficiency of lung and spleen qi which means there may existed some emotional-related disorders or hepatobiliary disease and the patient’s immune system was weak while the level of energy metabolism decreased. This prompted us to add Hedysarum Multijugum Maxim and Schisandrae Sphenantherae Fructus to replenish lung and spleen qi, and Radix Bupleuri to soothe the liver and dispel depression. In response, Hedysarum Multijugum Maxim and Schisandrae Sphenantherae Fructus were added to replenish lung and spleen qi, while Radix Bupleuri was included to soothe the liver and alleviate depression (Table 1D). Additionally, the dosage of the blood-cooling and detoxifying herb was subtracted accordingly.

**Figure 3.**
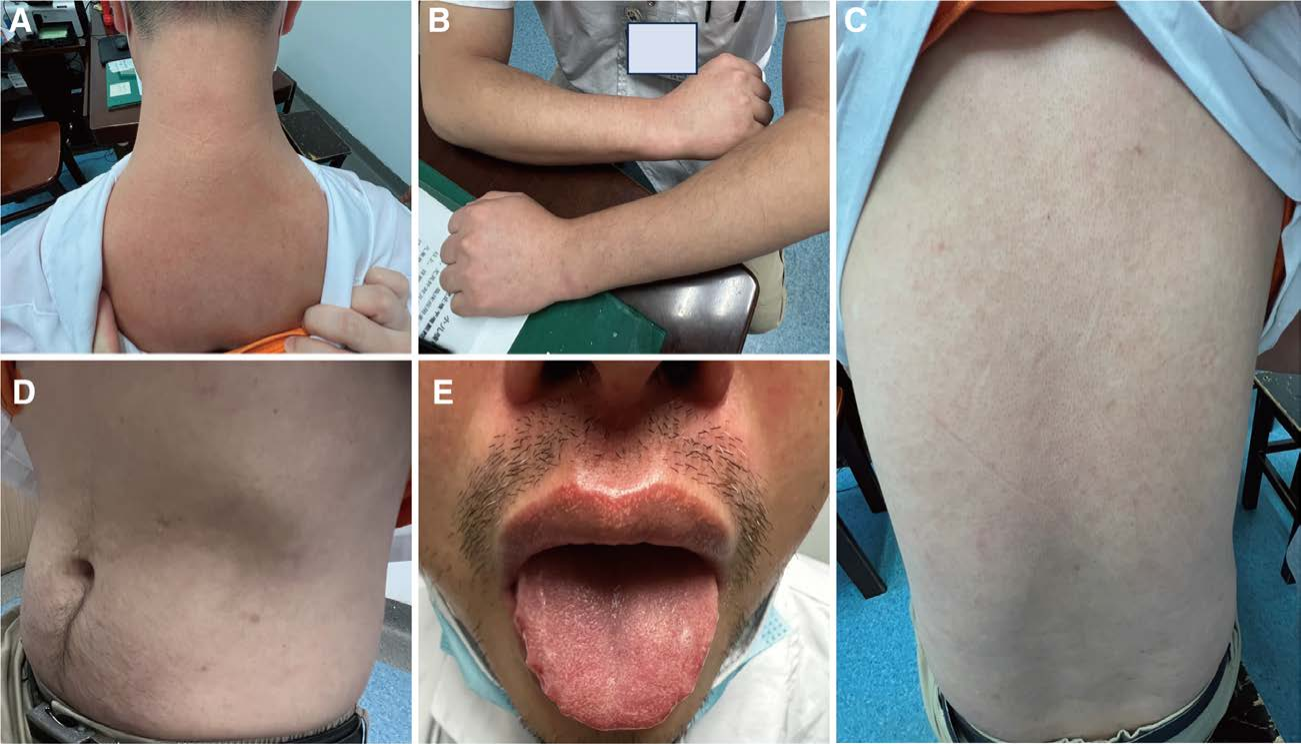
The patient’s skin condition at the beginning of September 2023 (A–E).

**Figure 4.**
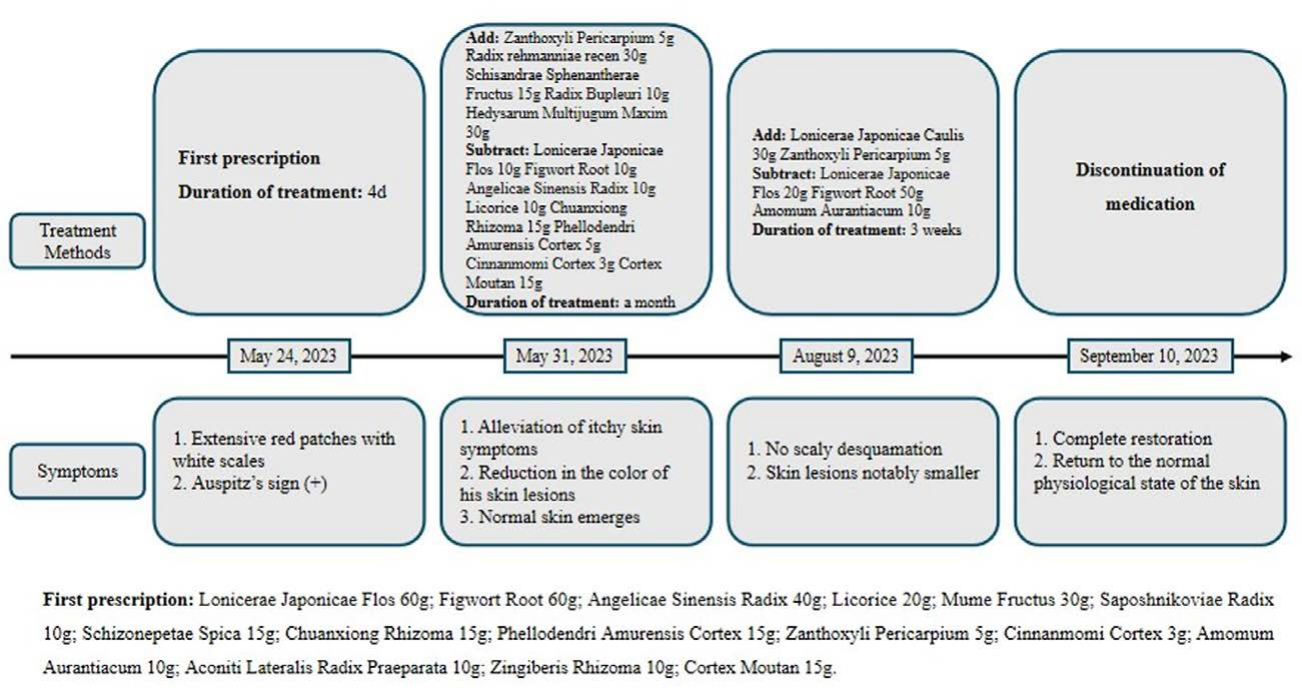
Timeline of symptom changes in the process of taking traditional Chinese medicine.

The patient rebuilt confidence and continued to take medication. Upon the third encounter on August 9, 2023, the patient no longer had scaly desquamation, and the skin lesions had become notably smaller than before (Fig. 2E–H). Over the subsequent two-month period spanning from May 31 to August, the patient maintained consistent utilization of the prescribed treatment and self-administered Chinese medicine. The patient’s final follow-up appointment in November revealed a desire to address the ongoing management of allergic rhinitis and blood sugar levels. During the consultation, the patient reported a complete healing and return to normalcy of his skin at the onset of September 2023. The tongue, a diagnostic tool in TCM, believed to reflect the patient’s physical condition and aid in treatment, exhibited improved clarity and appearance compared to previous assessments (Fig. 3A–E). As of the present, 8 months later, the patient’s skin condition has remained stable without any exacerbations. The patient expressed a renewed sense of vitality and well-being. For clarity and ease of understanding, a timeline chart has been included, correlating TCM treatment stages with psoriasis symptom improvement (Fig. 4).

### 2.3. Changes in the overall physical condition

It is noteworthy that the metabolic abnormalities, including blood lipids and blood glucose levels, associated with psoriasis have shown gradual improvement.^[23]^ Specifically, there was a decrease in low-density lipoprotein levels from 5.00 mmol/L to 4.12 mmol/L and a reduction in uric acid levels from 440 μmol/L to within the normal range. Daily physical examination reports once revealed the enlargement of both kidneys with foci of calcification in both kidneys and the prostate gland linked to psoriasis.^[24]^ We unexpectedly found these abnormalities were resolved without the use of any other Western medicine interventions. In conclusion, our analysis suggests that there were improvements in carbohydrate metabolism, lipid metabolism, and capillary microcirculation within skin lesions.

## 3. Discussion

When Yin and Yang are out of balance, it can lead to the onset of disease. WMW is considered an effective prescription for treating intermingled cold and heat syndrome, as it helps to restore physical functions and combat illnesses. This medication works by both warming the lower cold to enhance resistance and clearing the upper heat to restore equilibrium. WMW has been proven to inhibit intestinal inflammation and repair damaged intestinal mucosa.^[25]^ Restoring microbiota homeostasis and metabolic pathways, and reducing pro-inflammatory cytokine expression can alleviate symptoms in patients with psoriasis.^[26,27]^ This may be one of the mechanisms. Obesity may be an independent risk factor for psoriasis, white adipose tissue is the inflammatory basis of psoriasis and obesity. WMW prevents obesity by reducing white adipose tissue and enhancing brown adipose tissue function, which may be another pathway for treating psoriasis.^[28]^ By ensuring that the body’s qi and blood flow smoothly, optimal health can be achieved. In addition to medication, it is important to provide psychological counseling and advise patients to maintain a balanced diet and engage in regular exercise during treatment.

Psoriasis patients often have high blood viscosity and hyperviscosity, which can precipitate alterations in blood rheology and aberrations in blood composition, ultimately resulting in microcirculation disturbances, ischemia, hypoxia, and other related conditions that contribute to the progression of psoriasis.^[29]^ This phenomenon aligns with TCM theory, which posits that chronic illnesses can impair blood circulation and obstruct the flow of energy through the body’s meridians. Consequently, we should pay attention to help with blood flow in the management of psoriasis.

Psoriasis is a complex, multi-system disease. There is no one-size-fits-all solution for treating psoriasis until now. TCM offers unique advantages in addressing complex and intractable diseases through the use of pattern differentiation-based treatment. TCM compound prescriptions consist of a combination of herbs selected based on appropriate dosages and compatibility, under the guidance of TCM theory, and tailored to individual syndrome differentiations. This approach is characterized by its multi-component, multi-target, and multi-pathway functions.

## 4. Conclusion

The patient demonstrated strong adherence to TCM treatment exclusively in this case. TCM offers a cost-effective and efficacious approach to improving endocrine function and treating psoriasis. Nevertheless, further research, including additional case studies and randomized controlled trials, is necessary to substantiate the efficacy of TCM in treating psoriasis. The mechanisms underlying the therapeutic effects of Chinese herbs in this context remain ambiguous and warrant further investigation.

## Author contributions

**Conceptualization:** Miao Li.

**Formal analysis:** Miao Li.

**Investigation:** Lingjuan Liu.

**Methodology:** Lingjuan Liu.

**Resources:** Minhua Hu, Xiulan Dong.

**Supervision:** Minhua Hu.

**Visualization:** Zhilin Ge.

**Writing – original draft:** Lingjuan Liu.

**Writing – review & editing:** Xiulan Dong.
